# Machine Learning-Based Virtual Screening and Molecular Simulation Approaches Identified Novel Potential Inhibitors for Cancer Therapy

**DOI:** 10.3390/biomedicines11082251

**Published:** 2023-08-11

**Authors:** Muhammad Shahab, Guojun Zheng, Abbas Khan, Dongqing Wei, Alexander S. Novikov

**Affiliations:** 1 State Key Laboratories of Chemical Resources Engineering, Beijing University of Chemical Technology, Beijing 100029, China; shahabkhan1852@gmail.com; 2 Department of Bioinformatics and Biological Statistics, School of Life Sciences and Biotechnology, Shanghai Jiao Tong University, Shanghai 200240, China; abbaskhan@sjtu.edu.cn (A.K.); dqwei@sjtu.edu.cn (D.W.); 3 Institute of Chemistry, Saint Petersburg State University, Saint Petersburg 199034, Russia; 4 Research Institute of Chemistry, Peoples’ Friendship University of Russia (RUDN University), Moscow 117198, Russia

**Keywords:** CDK2, machine learning, virtual screening, molecular docking, MD simulation

## Abstract

Cyclin-dependent kinase 2 (CDK2) is a promising target for cancer treatment, developing new effective CDK2 inhibitors is of great significance in anticancer therapy. The involvement of CDK2 in tumorigenesis has been debated, but recent evidence suggests that specifically inhibiting CDK2 could be beneficial in treating certain tumors. This approach remains attractive in the development of anticancer drugs. Several small-molecule inhibitors targeting CDK2 have reached clinical trials, but a selective inhibitor for CDK2 is yet to be discovered. In this study, we conducted machine learning-based drug designing to search for a drug candidate for CDK2. Machine learning models, including k-NN, SVM, RF, and GNB, were created to detect active and inactive inhibitors for a CDK2 drug target. The models were assessed using 10-fold cross-validation to ensure their accuracy and reliability. These methods are highly suitable for classifying compounds as either active or inactive through the virtual screening of extensive compound libraries. Subsequently, machine learning techniques were employed to analyze the test dataset obtained from the zinc database. A total of 25 compounds with 98% accuracy were predicted as active against CDK2. These compounds were docked into CDK2’s active site. Finally, three compounds were selected based on good docking score, and, along with a reference compound, underwent MD simulation. The Gaussian naïve Bayes model yielded superior results compared to other models. The top three hits exhibited enhanced stability and compactness compared to the reference compound. In conclusion, our study provides valuable insights for identifying and refining lead compounds as CDK2 inhibitors.

## 1. Introduction

The cell division process is an essential event in life that involves an array of occurrences in a cell that results in the development of two identical daughter cells. It regulates the transition from cytokinesis to cell proliferation, and ensures genome stability through its checkpoints [1]. CDK2 interacts with and phosphorylates proteins in many different pathways, including DNA damage, intracellular transport, protein degradation, signal transduction, DNA and RNA metabolism, and translation. Cyclin-dependent kinase 2 (CDK2) drives the progression of the cell cycle, and is involved in DNA synthesis, G1 and S phase transition, and G2 phase modulation [2]. CDK2 is inactive in its monomeric form, similar to the CDKs; however, CDK2 is active when it forms a functional heterodimeric complex with one of its two regulatory partners, cyclins A and E, during the phosphorylation period. CDK2 modulates various oncogenic signaling pathways during phosphorylation of multiple transcription factors, including FOXM1, SMAD3, ID2, FOXO1, UBF, B-MYB, NFY, and MYC [3,4,5,6,7,8,9]. Moreover, the abnormal activation of CDK2 leads to uncontrolled cell proliferation during oncogenesis, and is critically associated with tumor growth in various human cancers [10]. Similarly, high levels of cyclin A and E regulatory subunits have been associated with oncogenesis of numerous cancers [11,12]. Overall, the activity of both CDK2 and the regulatory subunits is involved in the onset of the oncogenic process in human cancers. Despite the debated role of CDK2 in cancer development, recent studies indicate that targeting CDK2 specifically could offer therapeutic benefits in certain cancers. Therefore, pursuing CDK2 inhibition as a strategy in the development of anticancer drugs is still highly promising [13]. However, the efficacy of CDK2 inhibition in clinical settings has been limited by major challenges. The mechanism of CDK2 degradation is still unclear, and multiple CDK2 inhibitors produced in the past two decades lack the required specificity and are limited by off-target effects. Various small drug-like molecules have been progressed to preclinical and clinical trials as CDK2 inhibitors; however, a CDK2-targeted inhibitor still needs to be developed [14]. Recent technological advancements have entered a new era of precision drugs, leveraging data-driven disease assessment with machine learning (ML) in biomedical sciences. The use of AI-based approaches can be used to derive significant insights that then lead to better treatment outcomes. ML approaches are widely employed in cancer research and are gaining popularity in the identification and treatment of cancer [15]. ML-based techniques incorporate QSAR modeling, hit discoveries, and de novo drug designing to achieve accurate results [16]. The main goal of precision medicine in cancer research is to provide proven quality therapies with high survival chances of patients and reduced unwanted adverse effects [17]. These approaches have significantly assisted the analysis of large biomedical data in drug development and cancer therapeutic processes [18]. Moreover, the ML-based drug development approach is an accurate and effective method that optimizes lead compound identification and molecular understanding of a disease [19]. In this study, we focus on the identification of small drug-like molecules targeted to CDK2. This approach aims to provide the rationale for the therapeutic potential of CDK2-specific cancer treatment and the development of new-generation cancer therapeutics [20]. We used an integrated approach, namely, a ML-based virtual screening approach, to screen libraries of small molecules that have the potential to inhibit the activity of various CDK2-related oncogenic pathways. The shortlisted compounds with high druggable properties were further subjected to molecular docking and molecular dynamic simulation. These analyses evaluate the binding potential and molecular stability of these compounds in the active site of CDK2. The lead compounds proposed in this study could pave a path towards the development new precision drugs in cancer treatment. 

## 2. Material and Methods

### 2.1. Dataset Preparation

**Figure 1:**
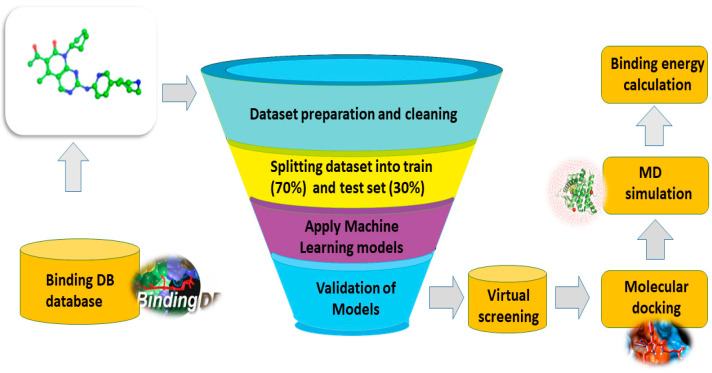
Schematic flow of the study. The current study involves 3D modeling, virtual screening, molecular simulation, and post-simulation analysis.

The dataset utilized in this study was obtained from BindingDB. A dataset consisting of 2277 compounds was obtained from the BindingDB database, which focused on CDK2 as a therapeutic target in cancer [21]. The compounds in the database were classified as “active” (1) or “inactive” (0). To prepare the data for analysis, data preprocessing and cleaning were conducted using the Python pandas package. The dataset was then divided into a training set (70%) and a test set (30%) for further analysis [22]. We divided the dataset into two sets: a training set, which accounted for 70% of the data, and a test set, which comprised the remaining 30% of the data. The next step involved data preprocessing, which encompassed three key tasks: data cleaning, data conversion, and feature extraction. The data cleaning process adhered to specific criteria: selecting only CDK2 inhibitors, choosing data with the biological activity value IC_50_, and removing duplicate compounds. Subsequently, a standard inhibitor for the target protein was sought, and based on the IC_50_ values of the standard compound, the dataset compounds were categorized as either active or inactive. In this study, compounds with IC_50_ values higher than the reference compound (Dalpiciclib) were considered inactive, while those with IC_50_ values equal to or below the reference compound were deemed active. Prior to conducting further analysis, the recurrent feature elimination (RFE) method, widely recognized for feature selection, was applied. The final dataset was used to train a random forest (RF) model [23], a support vector machine (SVM) model [24], Gaussian naïve Bayes (GNB) [25], and a k-nearest neighbor (k-NN) model [26]. The overall workflow of the work is given in Figure 1.

### 2.2. Descriptor Generation and Feature Selection

**Figure 2:**
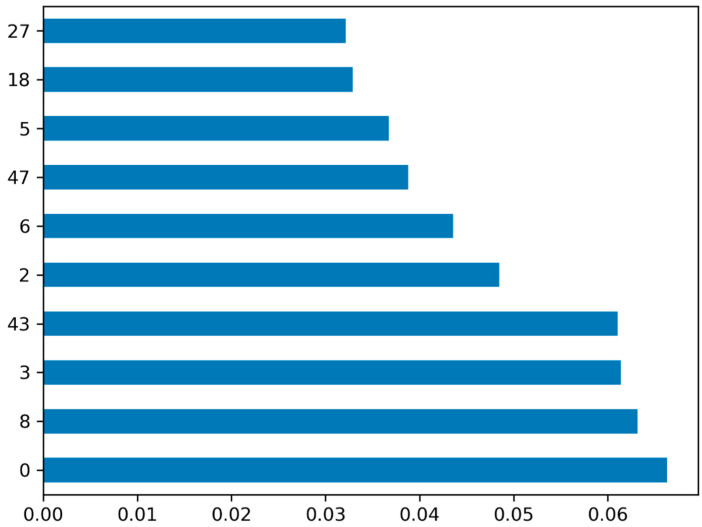
Optimum set of features selected from the training set.

A spreadsheet was created to store the 2D descriptors of 2277 compounds and ZINC library (containing 1lac) compounds, which were calculated using MOE 2022. The dataset underwent a recursive feature selection process to identify relevant features. This technique aims to improve the efficiency and effectiveness of machine learning algorithms by reducing the number of features, resulting in lower space and time complexity. Neglecting irrelevant input features is crucial, as they can negatively impact the prediction performance of certain machine learning algorithms. Throughout the preprocessing steps, the same training set was consistently used. To prevent overfitting and ensure unbiased results, constant features were removed and the feature set was normalized, preventing information leakage from the test set (Figure 2).

### 2.3. Machine Learning Algorithms

#### 2.3.1. SVM

A statistical supervised machine learning method called SVM was created by Boser and Vapnik, and it can identify recurring patterns in large datasets [27,28]. The data are structured as feature vectors, which exist in a space with the same dimensionality. This enables the construction of a hyperplane that separates the data points into two categories. The support vector machine (SVM) method is commonly employed in supervised learning, where the categories are predefined. However, SVM can also be utilized for unsupervised learning tasks [29]. Supervised learning is the preferred approach for virtual screening (VS) because it ensures that every compound will receive a definitive classification of either active or inactive from the classifier.

#### 2.3.2. k-Nearest Neighbor (k-NN)

In 1967, Cover and Hart designed a simple supervised machine learning approach to categorize patterns [30]. The algorithm operates by sorting query data points according to the votes received from the k-nearest data points. Various distance metrics, such as Euclidean distance, Hamming distance, cosine distance, and others, can be employed to determine the nearest neighbors.

#### 2.3.3. Random Forest

Random forest (RF) is a versatile method used for feature selection, clustering, regression, and classification problems. It consists of multiple trees generated from different bootstrap samples but the same distribution. An advantage of RF is that the collective prediction is unaffected by the high bias or variance of individual trees, making it a robust classifier. Majority voting in RF contributes to its strength as a classifier [31]. Weighting is incorporated into random forest (RF) to enhance the decision-making capability of individual trees. A tree with a higher error rate receives a lower weight value, enabling improved decision making. RF offers several advantages, such as high accuracy, effective performance with large datasets, estimation of important variables in classification, handling of missing data, and identification of outliers. These advantages contribute to the overall effectiveness and versatility of RF as a machine learning algorithm [32,33].

#### 2.3.4. Gaussian Naïve Bayes

Gaussian naïve Bayes (GNB) is a supervised learning method rooted in Bayes’ theory. It is a basic classifier that assumes each descriptor’s value is statistically independent in the naïve Bayesian classification. This algorithm considers the ratio of active to inactive by regarding the descriptor value as indicative of the probability of activity [34].

### 2.4. Performance Evaluation and Cross-Validation

To evaluate machine learning classification models, a confusion matrix is employed, providing a summary of properly predicted and misclassified observations. In previous approaches, efficient assessment metrics, such as accuracy, sensitivity, specificity, and MCC, have been utilized to measure the performance of the proposed model. The performances of the models were evaluated using these four parameters [35]. Accuracy, a commonly used assessment metric for classifiers, measures how well the classifier predicts TP (true positive) and TN (true negative) outcomes [36]. Here, the effectiveness of the model was evaluated using a statistical test known as 10-fold cross-validation.

### 2.5. Molecular Docking and Selection of the Final Hits

Active ligands against CDK2 and their binding interactions were identified using molecular docking. The crystal structure of the CDK2 complex (PDB ID: https://www.rcsb.org/structure/1B38, accessed on 1 June 2023) was retrieved from RCSB. The protein was first checked for any missing residues or atoms, and then prepared for docking. MOE v.2020 (molecular operating environment) software was utilized for protonation and energy minimization. The hits were then ranked based on their docking scores [37]. From a large number of docked conformations, only those that established contacts with critical residues in the active site were selected and evaluated. 

### 2.6. Molecular Dynamics Simulation (MDS)

A molecular dynamics (MD) simulation was conducted using the pmemd function from the AMBER-22 package to investigate the dynamics and interaction patterns of the top hits and reference complexes. To prepare the environment for simulation, one must generate the ligand topology files; for this, the antechamber module of the Amber-22 software was utilized. A 10 Å TIP3P water box was used to solvate the systems, and Na^+^ ions were added to neutralize each system. The protein force field ff19SB was used in this study [38]. All inhibitors were assigned Gasteiger charges, while the generalized AMBER force field (GAFF2) was employed as the force field for small molecules [28,39]. To maintain a temperature of 300 K, a Langevin thermostat was utilized, while a Berendsen barostat was employed to monitor a pressure of 1.0 bar for each system. A two-step energy minimization approach, involving steepest descent and conjugate gradient methods, was applied to optimize each system [40]. For calculating the long-range electrostatic interaction, we used the AMBER-22 Particle Mesh Ewald (PME) method. The cut-off distance for long-range electrostatic interactions and van der Waals interactions was 10Å. The covalent bonds were improved using the AMBER-22 SHAKE algorithm [41]. A total of three complexes of top ligand/CDK2 and one complex of control/CDK2 were subjected to MD simulation using the GPU version of AMBER-22, and the trajectory data from each simulated system were used in the analysis using the CPPTRAJ module of the AMBER-22 program. The graphical representation and binding interface analysis were completed using the MOE2022, PyMol, and Origin tools.

### 2.7. Binding Free Energy Calculation

MDS was used to create the trajectory, which was then utilized for calculating the binding free energy (BFE) using the MMPBSA.py script [42]. Numerous studies have used this method to figure out the binding free energy for protein–ligand, protein–protein, and protein–nucleic acid complexes. The total free energy of binding, denoted by the symbol Gbind, was determined with the assistance of the following equation:

∆G_bind_ = ∆G_com_ − (∆G_rec_ + ∆G_ligand_) (1)

∆G_bind_, ∆G_com_, ∆G_rec_, and ∆G_ligand_ represent the total binding energy, and the binding energies of complexes, proteins, and drugs, respectively. The individual binding energies that make up the overall binding free energy, such as those that are bonded (Gbond), electrostatic (Gele), polar (Gpol), and nonpolar (Gnpol), were estimated using the following equation.

(G = Gele + Gbond + GvdW + Gnpol + Gpol − TS)(2)

The binding free energies of the top compounds in complexes with CDK2, as well as the control complexes, were calculated using the molecular mechanics/generalized born surface area (MMGBSA) approach. The MMGBSA.py Python software in AMBER-22 was utilized for this purpose, enabling the determination of the binding free energies for the top hits and control complexes [43,44]. For the complexes, a decrease in potential energy over 100 ns serves as evidence of system stability. 

## 3. Results and Discussion

**Figure 3:**
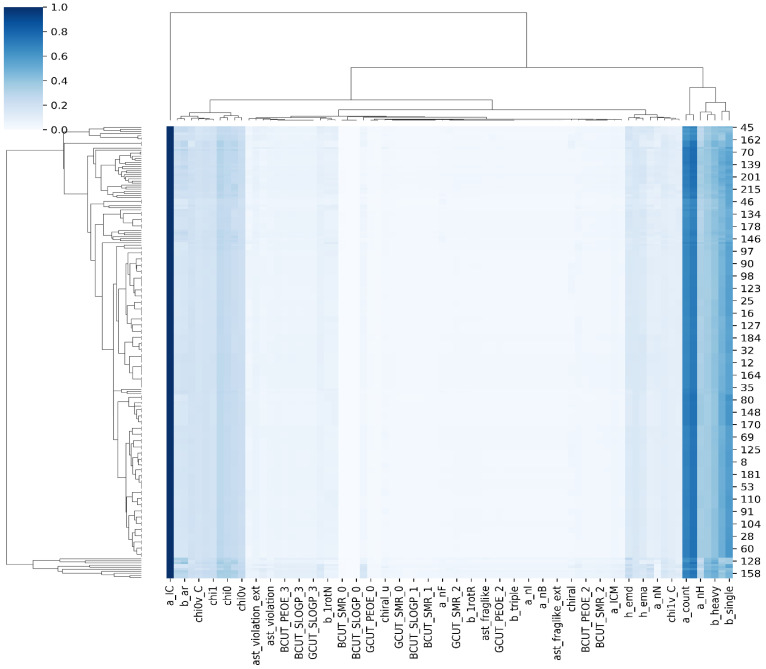
The CDK2 dataset heatmap created using Python’s Matplotlib tool.

**Figure 4:**
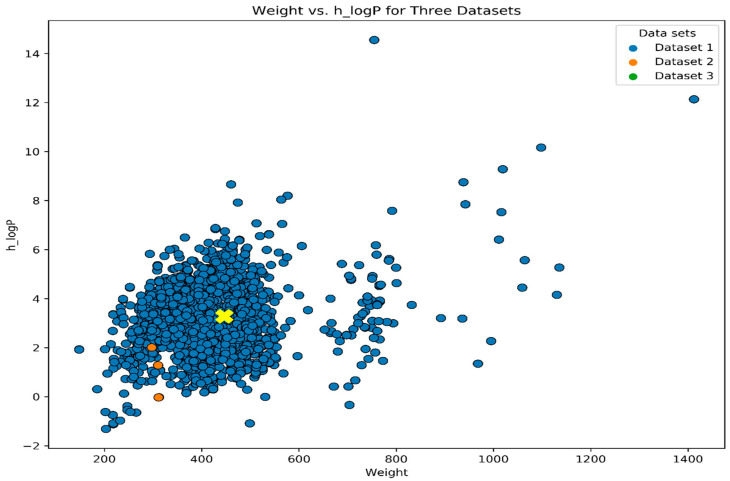
Diversity distribution of the training set and test. The chemical space is defined by the molecular weight and h_LogP.

In this study, we utilized k-NN, SVM, GNB, and RF machine learning algorithms, along with 207 2D descriptors computed using the MOE program. Data normalization was performed using the conventional Python scalar approach. The dataset was cleaned using the Python pandas package, resulting in a reduction in the 2D descriptors to 100. A popular visualization technique used in this study was the heatmap [43]. To visualize the data, we employed Python’s Matplotlib and seaborn packages. The interactive heatmap function of Python was used for data visualization. By utilizing the cluster heatmap, we were able to identify significant patterns within the dataset. Cluster heatmap is a powerful data visualization technique commonly used in various fields, including drug design, to identify patterns and relationships within a dataset. Similar compounds were clustered together based on their properties or activities, allowing for the observation of common structural features or activity trends that may be relevant to the interaction with the CDK2 target (Figure 3). Furthermore, the cluster heatmap enabled the prioritization of compounds for further investigation. Through visual inspection, clusters or groups of compounds exhibiting favorable properties, such as high inhibitory activity against CDK2, were identified. This facilitated the selection of potential lead compounds for subsequent experimental validation. The RFE (recursive feature elimination) method was employed to identify the relevant features in the dataset, which were subsequently utilized for model creation. By employing recursive feature elimination, the dataset was effectively simplified, and only the most relevant features were retained for model training. This helped to reduce the risk of overfitting, improve the model’s generalization to new data, and enhance interpretability. The dataset was divided into a training set (70%) and a test set (30%), as illustrated in Figure 4.

Several statistical metrics, including precision, recall, F1 score, and MCC, were utilized to evaluate the performance of the constructed models. The scoring function in traditional virtual screening often yields numerous false positive outcomes due to its limited ability to accurately predict protein–ligand binding. To enhance prediction accuracy, AI-based techniques have been implemented in the drug development process. AI strategies such as naïve Bayes, support vector machine (SVM), random forest (RF), and feed-forward artificial neural networks have demonstrated superior performance in the detection of new active compounds. Out of these models, the random forest (RF) achieved an accuracy of 89% and the highest MCC value of 0.893, surpassing other models, including k-NN. The k-NN model achieved an accuracy of 74% and an MCC value of 0.60. Overall, all the developed models exhibited commendable accuracy levels. Table 1 presents an overview of the performance of the four developed models, where the accuracy of Gaussian naïve Bayes (GNB) was 98%, and support vector machine (SVM) achieved an accuracy of 94%. However, in binary classification, the Matthews correlation coefficient (MCC) holds greater reliability, and provides more informative insights compared to accuracy. Consequently, the significance of all models was assessed based on the MCC value. Among the models, the random forest (RF) models displayed the highest MCC value and accuracy, making them the top performers. Following RF, the k-nearest neighbor (k-NN) model demonstrated the second-best performance in terms of both accuracy and MCC. GNB performed better than SVM in terms of both accuracy and MCC.

### 3.1. The ROC Curve of Each Algorithm

**Figure 5:**
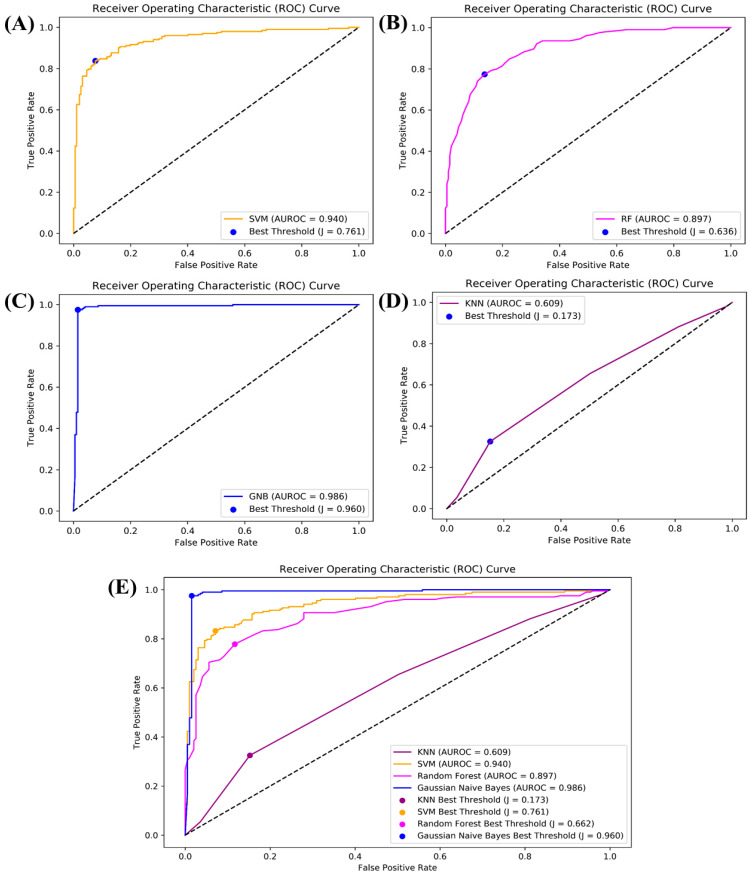
Illustrations of the ROC curves for each algorithm, indicating the accuracies of the ML models. (**A**) ROC curve for SVM, (**B**) ROC curve for RF, (**C**) ROC curve for GNB, (**D**) ROC curve for k-NN, (**E**) ROC curve for all algorithms.

In current study, four various machine learning algorithms, including RF, k-NN, GNB, and SVM, were used. By using MOE Version 2022, a total of 207 2D descriptors were calculated. In Python, a standard scalar technique was implemented, which was further used for data normalization. In order to clean the dataset, the Python pandas library was utilized, which resulted in 100 2D descriptors. Furthermore, the developed models were examined with the help of different statistical parameters: recall, MCC, F1 score, and precision. Nowadays, the scoring function for traditional virtual screening is not a precise screening approach to predict the binding of ligand and protein, and mostly results in a large set of false positive results. Therefore, AI-based approaches were utilized for drug designing. SVM, k-NN, RF, and GNB are important AI strategies for identifying the top active compounds. Additionally, we calculated Youden’s index, which is also known as the J statistic or Youden’s J; this is a single summary statistic that combines both sensitivity and specificity to evaluate performance. In the context of ROC curves, Youden’s index helps identify the threshold that corresponds to the point on the curve where the sum of sensitivity and specificity is maximized, often referred to as “Youden’s J point”. Plotting this point on the ROC curve can be helpful in assessing the performance of a classifier and selecting an appropriate threshold for practical applications. The accuracy of k-NN was 0.609%, Youden’s index was 0.172839, and the best threshold value was J = 0.173, whereas the accuracy of RF was 0.897%, Youden’s index was 0.567228, and the best threshold value was J = 0.636. The accuracy of SVM was 0.940%, Youden’s index was J = 0.761, and the best threshold value was 0.649143, whereas for GNB, the accuracy was 0.986%, Youden’s index was J = 0.960, and the best threshold value is 0.085291, respectively. The ROC curve (AUC) was formed in Python, and is illustrated in Figure 5. The ROC curve identify the presence of non-hits or hits for a given protein. The ROC curve provides a visual tool that compares the performances of various algorithms, and selects the one that balances the specificity and sensitivity for a given task. The area under the ROC curve (AUC) was utilized as a summary statistic, comparing the overall performances of various algorithms, with higher values of AUC representing better performance. To evaluate the binary classifier performance, we utilized ROC curves that displayed false positive rates and true positive rates at different threshold values. It can also be utilized to summarize the classifier performance with a single numerical value by comparing it with different classifiers. The area under the ROC curve is an important metric utilized for such a purpose.

### 3.2. Binding Interactions of Final Selected Hits

**Figure 6:**
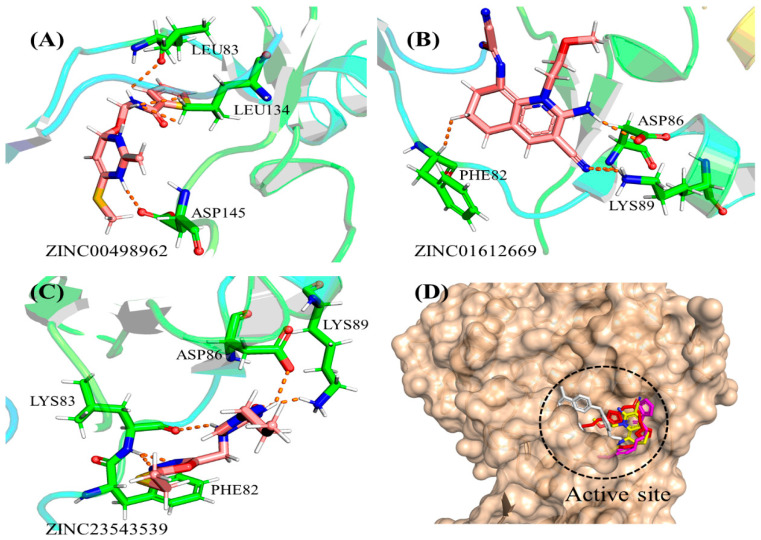
(**A**–**D**) The binding modes of the final selected hits within the binding site of CDK2.

To enhance the quality of the retrieved hit compounds, molecular docking was employed using a molecular docking tool. This process involved docking all initially retrieved hits and a control compound into the active region of the CDK2 protein. The goal was to refine and assess the binding interactions of these compounds. During the docking studies, it was observed that the three finalized hit compounds contained electronegative functional groups, which facilitated significant polar and hydrophobic interactions. The docking simulations conducted using MOE 2020 indicated that potent anticancer drugs exhibited strong hydrogen bonding and hydrophobic interactions with critical receptor residues of the CDK2 protein. The docking results of the selected compounds with CDK2 provided useful information about the nature of the binding mode, which was strongly correlated with the experimental results. From the docking studies, it was observed that compound ZINC00498962 produced a good docking score (−8.00) among the selected hit compounds. Good binding energy and binding affinity for this compound were observed compared to other hits. The docking conformation of this compound showed that it formed three hydrogen bonds with the active site residues Asp 145, Leu 134, and Leu 83 (Figure 6A). Asp 145 was observed to form a hydrogen bond with the hydrogen atom of the hydroxyl group, whereas Leu 134 formed a hydrogen bond with the oxygen atom of the hydroxyl moiety of the compound, and this compound was mapped very well on the pharmacophore features. Furthermore, the compound ZINC01612669 also demonstrated a good binding interaction score, i.e., −7.90, forming a total of four hydrogen bonds. The binding mode of this compound showed that it formed interactions with the active site residues Leu 83, Asp 86, Lys89, and Phe 82 (Figure 6B). Asp 83 formed a hydrogen bond with the hydrogen atom of the –NH moiety of the compound ZINC01612669. The benzene ring of 1,1′-biphenyl was observed forming an arene cation interaction with Lys 432. Arg 476 was observed forming a polar interaction with the carbonyl oxygen moiety of the compound. Likewise, compound ZINC23543539 exhibited a good docking score (−7.34) compared to the control drug (Figure 6C). The docking conformation of ZINC23543539 showed that Leu 83 formed a hydrogen bond with the –NH moiety atom of the compound. The significant potencies of the retrieved hit compounds may be attributed to several factors, including the presence of electron-donating groups (–OH and –NH), electron-withdrawing groups (–nitro), and the electron cloud density within the six rings (delocalized electrons). These characteristics render the hit compounds potent and polarizable, contributing to their observed potency. When compared to the reference compound, all of the finalized hit compounds exhibited positive interactions with the CDK2 protein, as well as favorable docking scores. The docking scores of all the lead compounds are listed in Table 2. 

### 3.3. Molecular Dynamics Simulation Analyses

#### 3.3.1. Root Mean Square Deviation (RMSD) Analyses 

In order to identify the well-stabilized and equilibrated structures of protein–ligand complexes, a 100 ns MDS was carried out using the AMBER-22 software. For instance, more stable binding of a ligand to a protein’s active pocket is linked to greater pharmacological potential than comparatively unstable binding. The stability of a simulation trajectory can be demonstrated by using the RMSD function integrated with simulation tools. So, in order to determine whether these compounds are stable during simulation, we also calculated RMSD for these trajectories as a function of time. The MDS results suggest three drugs (ZINC ID: ZINC00498962, ZINC01612669, and ZINC23543539) that could be the inhibitors of CDK2.

**Figure 7:**
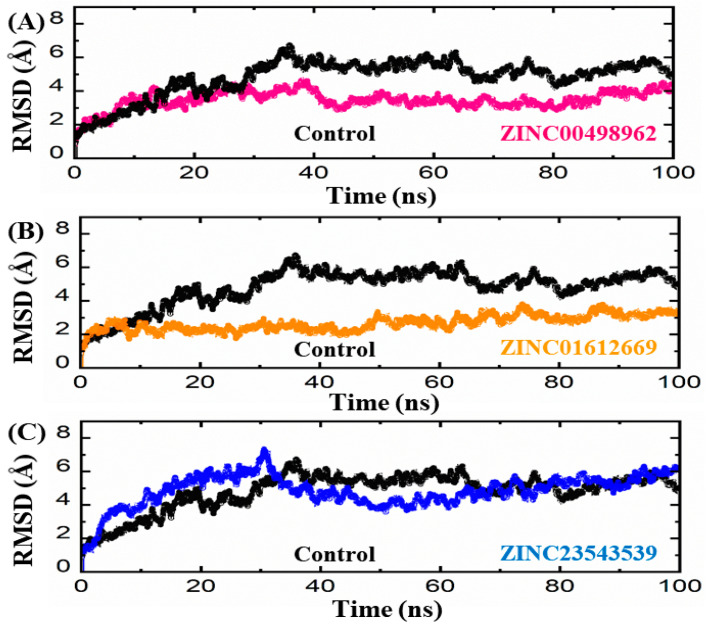
(**A**–**C**) RMSD plots of the repurposed drugs/CDK2 and reference drug (control). The *x*-axes and *y*-axes show the time in nanoseconds (100 ns) and RMSD in Angstroms, respectively.

The result of the MD simulation suggests that, as compared to the reference compound, the root mean square deviation (RMSD) of ZINC00498962 in complex with the receptor converged after 40 ns of the simulation, and remained stable throughout the simulation period, with an average RMSD value of 2.5 Å (Figure 7A). The CDK2 in complex with ZINC01612669 also converged after 5 ns of simulation, and could remain stable during the entire simulation period, with an average value of 1.5 Å, and with a slight deviation at the start of the simulation (Figure 7B). Moreover, the result of MD simulation suggests that, as compared to the reference compound, the root mean square deviation (RMSD) of CDK2 in complex with ZINC23543539 converged after the 30 ns of simulation, and then retained stable throughout the simulation period, with an average value of 1.15 Å (Figure 7C). The superimposition of all three simulated systems (reference, ZINC00498962, ZINC01612669, and ZINC23543539) suggests that ZINC01612669 behaved more stably, followed by ZINC00498962 and ZINC23543539, as compared to the reference compound of CDK2.

#### 3.3.2. Root Mean Square Fluctuation Analyses

**Figure 8:**
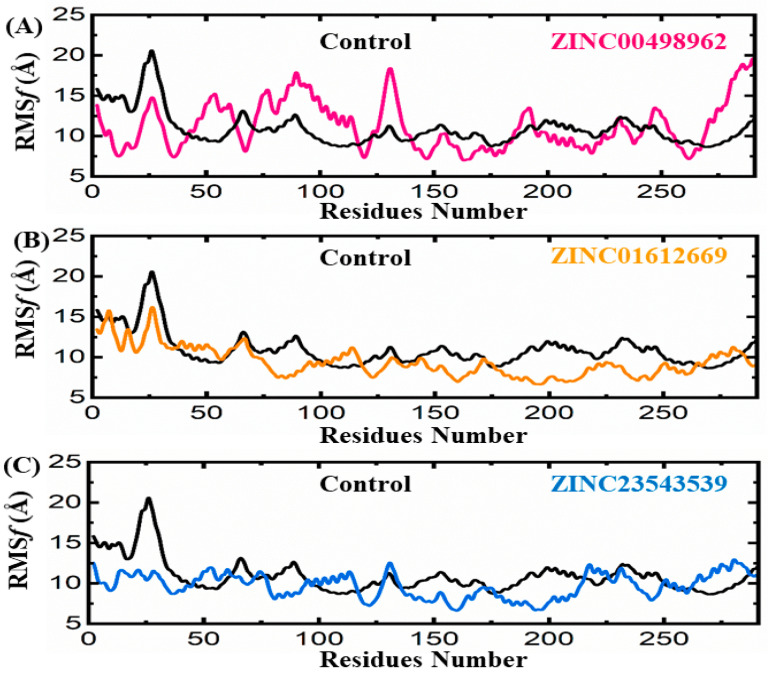
(**A**–**C**) RMSF plots of the repurposed drugs/CDK2 and reference drug. The *x*-axes and *y*-axes show the residual number and RMSF in Angstroms, respectively.

Root mean square fluctuation (RMSF) is an important parameter that describes the flexibility of each residues of the receptor. The RMSF analyses reflect the stability of the receptor in general and the fluctuation of the binding pocket residues in particular. The results of our RMSF analyses suggest that the control compound of the CDK2 receptor showed high fluctuation (average value ~14 Å) as compared to the fluctuation observed for the complex of CDK2 and ZINC00498962 (average value ~13.5 Å) (Figure 8A). In parallel, the ZINC01612669 complex exhibited the lowest RMSF (average value ~9 Å) with the CDK2 receptor (Figure 8B). Furthermore, the RMSF analyses of CDK2 and the candidate inhibitors suggest that the reference compound showed high fluctuation (average value ~14 Å) as compared to the fluctuation observed for the complex of CDK2 and ZINC23543539 (average value ~8.5 Å) (Figure 8C). The comparative analyses suggest that of the three simulated systems (ZINC00498962, ZINC01612669, and ZINC23543539), ZINC23543539 behaved more stably, followed by ZINC00498962 and ZINC01612669, as compared to the reference compound of CDK2.

#### 3.3.3. Radius of Gyration

**Figure 9:**
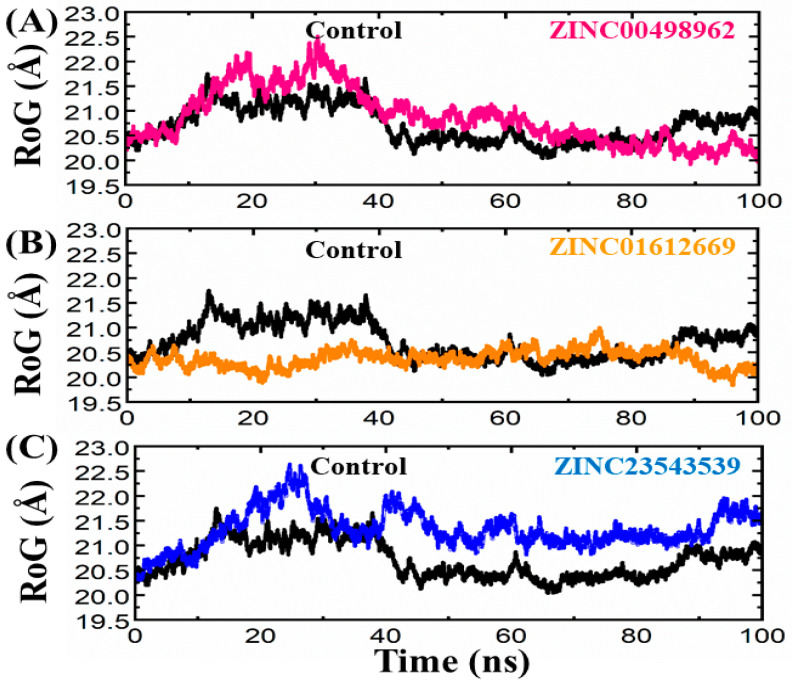
(**A**–**C**) RoG plots of the repurposed drugs/CDK2 and reference drug. The *x*-axes and *y*-axes show the time interval and RoG in Angstroms, respectively.

To assess the compactness of the system, a plot was generated to examine the relationship between RoG and time. Lower RoG values indicate a highly stable and compact structure in relation to conformational entropy, while higher RoG values suggest a less compact structure. Figure 9 illustrates the simulation RoG values for the lead hit compound, the control drug, and others. In this analysis, we examined the relationship between the radius of gyration (RoG) and time to understand the compactness of the structure. The control drug complex showed variations in RoG during different time intervals, consistent with the RMSD data. RoG slightly increased between 20 and 40 ns, but then stabilized for the remainder of the 100 ns simulation. The average RoG of this complex was determined to be 20.5 Å. In contrast, the ZINC00498962 complex exhibited deviations at various time intervals, despite having a similar RoG value. This trend persisted throughout the first 20 to 30 ns of the simulation. The average RoG for this wild-type complex was calculated to be 21.0 Å. Furthermore, the ZINC23543539 complex demonstrated a consistent pattern of RoG values throughout the simulation. There was a minor decrease in RoG between 20 and 25 ns, followed by stabilization until the completion of the 100 ns simulation. The average RoG for this wild-type complex was determined to be 20.2 Å. However, ZINC01612669 exhibited a fluctuating pattern of RoG values throughout the simulation. There was a continuous increase and decrease in RoG between 10 and 65 ns, followed by a subsequent increase until 100 ns. The RoG value remained relatively lower for the remaining simulation time, although significant deviations were observed. The RoG graphs for each complex can be seen in Figure 9A–C.

#### 3.3.4. Hydrogen Bond (Polar Interactions) Analyses

**Figure 10:**
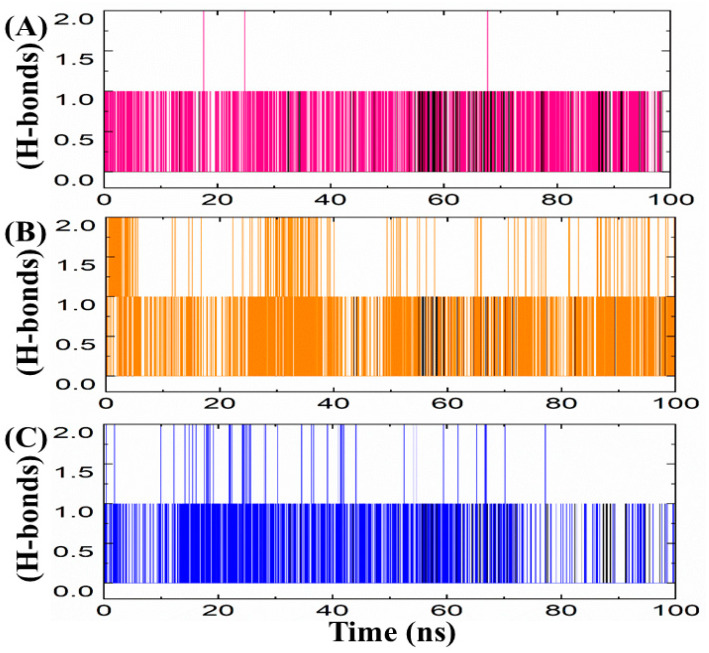
(**A**–**C**) Hydrogen bonding plots of the repurposed drugs/CDK2 and reference drug. The *x*-axes and *y*-axes show the time in ns and the number of hydrogen bonds formed, respectively.

Hydrogen bond analysis is an important parameter to understand the binding affinities and stabilities of protein–ligand complexes in drug discovery platforms [45]. Herein, the simulation results of our study showed that ZINC01612669 retained a single hydrogen bond with the binding pocket residues of CDK2 in the middle of the simulation over a period of almost 20 ns, as shown in Figure 10A. On the other hand, ZINC00498962 retained a single hydrogen bond with CDK2 during the entire simulation period (Figure 10B). In contrast, ZINC23543539 established and retained a single hydrogen bond with CDK2 over the entire simulation period, as shown in Figure 10C. The superimposed views of the hydrogen bond patterns of the reference compound, ZINC23543539, ZINC01612669, and ZINC00498962, with the binding pocket residues of CDK2, are displayed in Figure 10.

#### 3.3.5. Clustering of Protein Motion by PCA

**Figure 11:**
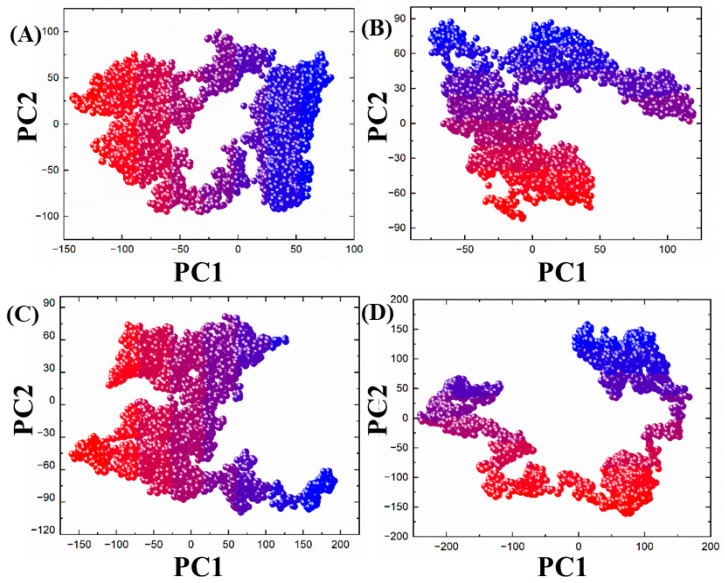
(**A**–**D**) Representations of the protein clustering analysis: (**A**) ZINC00498962, (**B**) ZINC01612669, (**C**) ZINC23543539, (**D**) Dalpiciclib drug (control).

Here, we describe how we utilized PCA in order to cluster the protein motion, and to explore the conformational changes in the target and the chemistry of the bonded compounds during the MD simulation period. The two PCs (PC1 and PC2), which reflected the motions in two dimensions, were used. For each complex, distributed principal components are provided in Figure 11A–D. It is noted that ZINC00498962 and ZINC01612669 presented more local motions, and wider distributions of protein clusters, separated through subspace from blue to red. The dots of ZINC00498962 were found to be arranged and compact as compared to ZINC01612669, starting from red dots and ending in blue dots, covering an area of −145 and +60 along PC1 and −100 and +100 along PC2, while in ZINC01612669, covering an area of −55 and +120 along PC1 and −70 and +90 along PC2 over a 100 ns trajectory. In contrast, the PCA graph for ZINC23543539, covering an area of −150 and +200 along PC1 and −100 and +90 along PC2, presented a higher distribution, as illustrated in Figure 11C. On the other hand, the Dalpiciclib drug showed a wide distribution and an unstable binding pattern of compounds into the binding sites of check point kinase (Figure 11D). The results indicate that almost all compounds have similar patterns of motions, which were presented during the MD simulation time. Hence, the compound residues are at the same site, resulting in the same energy state. 

#### 3.3.6. Free Energy Landscape

**Figure 12:**
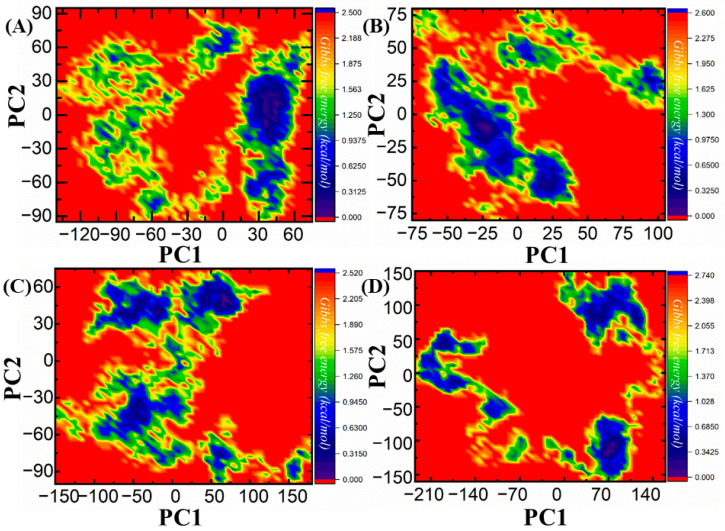
(**A**–**D**) Representations of the free energy landscape: (**A**) ZINC00498962, (**B**) ZINC01612669, (**C**) ZINC23543539, (**D**) Dalpiciclib drug (control).

Here, we evaluate the free energy landscape by using the principal component analysis, which represents more a reliable presentation of the energy- and time-dependent conformation space of proteins. Subsequently, the free energy landscape approach differentiates between thermodynamic and kinetic characteristics of proteins, which makes it a more reliable tool for deeply studying the dynamic property of proteins. As illustrated in Figure 12, the FEL plots for both PC1 and PC2 present fluctuations in the form of Gibbs free energy from red to blue, which indicates a protein shift from an unstable high-energy phase into a stable and lower-energy phase. The analysis of FEL demonstrates the conformational dynamics of PC1 and PC2 of the Dalpiciclib drug and ZINC00498962, ZINC01612669, and ZINC23543539. The zinc complexes, i.e., ZINC00498962, ZINC01612669, and ZINC23543539, attained low energy conformations and more stable behaviors as compared with the Dalpiciclib drug. Hence, the overall results reveal dynamic and conformation changes that favor the binding of these inhibitors more so than the Dalpiciclib drug. 

#### 3.3.7. Binding Free Energy Calculation

The molecular mechanics/generalized born surface area (MMGBSA) method is primarily used for rescoring the docked pose of a ligand. These poses are taken as inputs for the energy minimization for the protein–ligand complexes. It generates different ligand orientations by using various docking software, which further employs it for generating ligand–receptor coordinates for MD simulation analysis. The best binding free energy is obtained and the most reliable ligand review version orientation is identified. Moreover, it also analyses the contribution of each receptor residue interacting with the ligand and evaluates the individual energy terms of the binding free energy. Therefore, by using the MMGBSA method, it is possible to develop an in-depth analysis paradigm for receptor–ligand interactions and eventually design new active ligands. It was predicted that the final selected ZINC00498962/CDK2 complex, ZINC01612669/CDK2, and ZINC23543539/CDK2 had even lower MMGBSA scores of −24.1515 ± 2.1687 Kcal/mol, −32.7382 ± 2.5079 Kcal/mol, and −20.1473 ± 3.1713 Kcal/mol, respectively, while the control/CDK2 complex had a score of −19.7267 ± 3.4638. The results were correlated with the docking scores to design relevant drug-like potent inhibitors because the value ΔG bind defines the greater binding affinity of the ligand to the receptor. The outcome of MMGBSA was most supportive in understanding the binding mode analysis of the ligand with the receptor to help in the generation of potent inhibitors against CDK2.

## 4. Conclusions

Cyclin-dependent kinase 2 (CDK2) is considered a promising drug target for cancer therapy. In this study, the zinc database was screened against CDK2 using machine learning algorithms, and 25 compounds were ultimately predicted to be active against CDK2 with an accuracy rate of 98%. All of the predicted compounds were subsequently docked onto the CDK2 active site. Finally, the top three hits were selected based on good docking score, and good binding interactions were subjected to MD simulation in order to better understand the dynamic behavior of the discovered hits. Additionally, the Dalpiciclib/CDK2 complex was simulated as a control. As compared to the control drug Dalpiciclib, the three predicted hit compounds, namely, ZINC00498962, ZINC01612669, and ZINC23543539, showed consistent behavior and compactness, and the more flexible regions were seen in the control, with slight changes. Despite the promising findings, this study has several limitations, and future research should carefully consider data biases and potential overfitting when applying machine learning algorithms. Additionally, molecular docking simulations may be sensitive to various parameters and choice of software, warranting caution in interpreting docking scores as definitive indicators of compound activity. Furthermore, the study would greatly benefit from conducting in-depth in vitro and in vivo clinical testing to validate the inhibitory activities and potential therapeutic efficacies of the predicted compounds against CDK2. Additionally, exploring the impacts of the compounds on off-target interactions and toxicity profiles is crucial in drug development.

## Figures and Tables

**Table 1:** Performances of machine learning models.

Algorithms	F1_Score	ACC	roc_auc Score	Specificity	Sensitivity
SVM	0.753	0.940	0.940	0.998	0.489
k-NN	0.653	0.609	0.743	0.998	0.489
RF	0.653	0.893	0.893	0.998	0.489
GNB	0.93	0.986	0.888	0.992	0.482

**Table 2:** Protein–ligand interaction details and the docking scores of the standard drug and final hits.

Compound	Score	Interaction Details							
		Ligand		Receptor			Interaction	Distance	E (kcal/mol)
ZINC00498962	−8.00	N1	3	OD1	ASP	145	H–donor	3.25	−2.5
		S1	17	OD2	ASP	86	H–donor	3.72	−2.5
		C12	19	OD2	ASP	145	H–donor	3.41	−0.2
		O2	12	CD2	LEU	134	H–acceptor	3.47	−0.1
		S1	17	CD2	LEU	134	H–acceptor	3.05	−0.2
		N3	10	6-ring	PHE	82	H–pi	3.47	−1.2
		C9	14	6-ring	PHE	82	H–pi	4.49	−0.1
		5-RING		NZ	LYS	89	pi–cation	4.34	−0.5
ZINC01612669	−7.90	C10	12	O	LEU	83	H–donor	3.26	−0.3
		N2	15	OD2	ASP	86	H–donor	3.43	−1.8
		N3	17	CA	GLN	85	H–acceptor	3.63	−0.3
		N3	17	NZ	LYS	89	H–acceptor	3.09	−7.6
		N6	23	N	ASP	145	H–acceptor	3.82	−0.4
		C8	10	6-RING	PHE	82	H–pi	4.51	−0.4
ZINC23543539	−7.34	N4	9	OD2	ASP	86	H–donor	3.19	−2.4
		N5	12	O	LEU	83	H–donor	3.56	−0.6
		S1	19	O	GLU	81	H–donor	3.77	−1.3
		O1	11	NZ	LYS	89	H–acceptor	3.26	−3.7
		N6	15	N	LEU	83	H–acceptor	3.38	−1.1
		N7	16	CA	PHE	82	H–acceptor	3.55	−0.5
		N7	16	N	LEU	83	H–acceptor	3.35	−0.8
Control drug (Dalpiciclib)	−6.34	N23	23	OD1	ASP	145	H–donor	2.64	−9.7
		O1	1	NZ	LYS	129	H–donor	3.37	−1.7
		O32	32	NZ	LYS	129	H–acceptor	2.77	−0.5
		6-RING		CG	GLN	145	pi–H	3.17	−2.2

## Data Availability

All data generated and analyzed during this study are included in the article.
